# Proteomic Analysis Reveals Cadherin, Actin, and Focal Adhesion Molecule-Mediated Formation of Cervical Cancer Spheroids

**DOI:** 10.3390/cells13232004

**Published:** 2024-12-04

**Authors:** Piyatida Molika, Kittinun Leetanaporn, Wararat Chiangjong, Pongsakorn Choochuen, Raphatphorn Navakanitworakul

**Affiliations:** 1 Department of Biomedical Sciences and Biomedical Engineering, Faculty of Medicine, Prince of Songkla University, Songkhla 90110, Thailand; piyatidamolika@gmail.com (P.M.); lkittinun1@gmail.com (K.L.); am_pcc1@hotmail.com (P.C.); 2 Pediatric Translational Research Unit, Department of Pediatrics, Faculty of Medicine Ramathibodi Hospital, Mahidol University, Bangkok 10400, Thailand; wararat.chi@mahidol.ac.th

**Keywords:** cell culture methods, 3D culture, 2D culture, cancer cell lines, cervical cancer

## Abstract

Cancer spheroids are spherical, three-dimensional (3D), in vitro assemblies of cancer cells, which are gaining importance as a useful model in cancer behavior studies. Designed to simulate key features of the in vivo tumor microenvironment, spheroids offer reliable insights for drug screening and testing applications. We observed contrasting phenotypes in 3D cervical cancer (CC) cultures. Thus, in this study, we compared the proteomes of 3D and traditional two-dimensional (2D) cultures of CC cell lines, HeLa, SiHa, and C33A. When cultured in in-house poly-(2-hydroxyethyl methacrylate)-coated plates under conditions suitable for 3D spheroid formation, these CC cell lines yielded spheroids exhibiting different features. Proteomic analysis of cells cultured in 2D and 3D cultures revealed similar protein profiles but remarkable differences in the expression levels of some proteins. In SiHa and C33A cells, the upregulation of key proteins required for spheroid formation was insufficient for the formation of compact spheroids. In contrast, HeLa cells could form compact spheroids because they upregulated the proteins, including cadherin-binding, cytoskeleton, and adhesion proteins, necessary for spheroid formation during the remodeling process. Overall, this study unravels the mechanisms underlying the formation of spheroids in the commonly used CC cell lines.

## 1. Introduction

Cervical cancer (CC) is the fourth most common cancer and a major cause of cancer-related deaths in females worldwide [1]. Epidemiological studies have shown that sexually transmitted human papillomavirus (HPV) is involved in the development of CC.

Research in cell biology, tissue morphology, disease mechanisms, drug action, protein synthesis, and tissue engineering has primarily been conducted using two-dimensional (2D) cell cultures in vitro. However, 2D cultures have several limitations, including the inability to simulate the interactions of cells with their environment, alterations in cell structure and behavior, and changes in cell division patterns. The 2D monolayer culture of cells grown on a flat surface does not adequately represent the cells in their in vivo settings. In the body, cells and extracellular matrix are organized into specific three-dimensional (3D) structures. Conventional monolayer cell cultures fail to replicate the in vivo surroundings of cells, and consequently, experiments relying on such cultures fail to factor in crucial aspects, such as cell-to-cell and cell-to-matrix interactions, nutrient distribution, and physiological and biochemical characteristics. To overcome these drawbacks, advanced models, such as 3D spheroid cultures, have been developed that better replicate the in vivo conditions.

Three-dimensional spheroid technology has become an essential tool in cancer research, providing a model that closely resembles physiological conditions for studying human cancers. By accurately mimicking organ structure and function, spheroids offer a flexible platform for exploring various clinical and biomedical questions, including those related to pharmacology and disease mechanisms [2,3]. However, the use of 3D spheroids in cancer research has the following limitations: (i) they exhibit limited complexity compared to in vivo tumors; (ii) their small size limits their ability to replicate larger tumors, and reduces the diffusion of nutrients and oxygen with growth that can lead to necrosis of the core; (iii) the formation and standardization of spheroids is technically challenging; (iv) difficulty in imaging and analysis, requiring advanced techniques; and (v) limited suitability for certain high-throughput screening because of the complexity and time involved in creating and maintaining them. Despite these limitations, 3D spheroids provide significant advantages. The major advantages are as follows: (i) spheroids are physiologically relevant as they replicate the 3D structure of tumors and closely mimic the cell-to-cell and cell-to-extracellular matrix (ECM) interactions found in actual human tissues; (ii) gradients of nutrients, oxygen, and pH, similar to those in tumors, are naturally formed in spheroids. These gradients are essential for exploring the adaptation of cancer cells to different microenvironments and for testing drugs targeting hypoxic regions; (iii) spheroids can contain a mix of cell types, including fibroblasts and immune cells, better replicating the tumor microenvironment; (iv) because of their structural complexity, spheroids can be used to more accurately predict the effectiveness and toxicity of drugs compared to 2D cultures. This predictive power is crucial for preclinical drug screening, reducing the likelihood of false positives that might fail in animal or human trials; (v) spheroids offer a simpler, more ethical alternative to animal models in early-stage testing. They reduce the need for animal studies, which are often costly, time-consuming, and raise ethical concerns. Thus, while spheroids offer a more realistic and cost-effective model for early cancer research compared to 2D culture, they have certain limitations that should be considered, especially when translating findings to in vivo or a clinical setting [4,5,6,7,8]. The cancer spheroid model has become an essential tool in research on various cancer types, such as colorectal, ovarian, and breast cancers [9,10,11]. Colorectal cancer (CRC) cells can be grown as spheroids when seeded at low densities in a culture medium supplemented with growth factors [9]. However, Sargenti et al. reported differences in the physical characteristics of CRC spheroids in culture, even when starting from the same number of cells. Spheroids formed from CRC cell lines HCT-15, DLD-1, and SW620 showed round shapes with smooth surfaces, whereas those formed from other cell lines, such as HT-29, showed irregular shapes with rough surfaces [12].

In another study, inconsistent spheroid morphology was observed in 3D cultures of CC cell lines, including HeLa (HPV18+), SiHa (HPV16+), CaSki (HPV16+), and C33A (HPV-negative) [13]. Several studies have explored the use of HeLa cell spheroids in cancer research, leveraging their 3D structure to mimic tumor environments more accurately than 2D cultures. HeLa spheroids provide valuable insights into cancer cell growth, drug resistance, and invasion mechanisms, often demonstrating behavior closer to in vivo tumors than that possible using traditional cell cultures [13,14,15]. In a study, time-lapse imaging was used to track the proliferation of HeLa spheroids in soft agar, enabling precise observation of cell behavior and responses to drug treatment in a 3D setting [14]. This approach helps refine drug testing by offering sensitivity to changes within the spheroids, which conventional 2D cultures may not capture effectively. In another study, comparison of the characteristics of spheroids from various CC cell lines, showed that HeLa cells formed compact spheroids, mimicking the solid structure of tumors, that displayed biological properties relevant to tumor morphology and growth, allowing for more consistent high-throughput testing [13]. Additionally, HeLa spheroids have been incorporated into collagen matrices within microfluidic platforms, allowing researchers to examine invasion patterns. This setup mimics the ECM and enables control over fluid dynamics, making it ideal for studying directional invasion in 3D cancer models [15]. SiHa, a human cervical cancer cell line, can indeed form spheroids in 3D culture conditions. These spheroids generally exhibit a compact and round morphology, although their density and structure can vary depending on the culture method and conditions used. SiHa spheroids are often denser and more cohesive than those formed by some other CC cell lines, such as C33A, because of the specific adhesion properties and characteristics of SiHa cells, which carry HPV-16 [14,15]. C33A cells can form spheroids, whose morphology and compactness may differ from those formed by other CC cell lines. C33A spheroids typically exhibit a less compact, looser structure compared to HeLa or SiHa spheroids, which can result in a more irregular or dispersed morphology. This difference may stem from the intrinsic properties of C33A cells, which are not HPV infected and express unique adhesion and proliferation characteristics that impact their aggregation in a 3D model. Although C33A cells can form spheroids under low-adhesion conditions or when cultured in certain 3D matrices, their spheroids are often less uniform and may display a more porous or heterogeneous appearance. This looser morphology may affect the response of these cells to drugs or their interaction with immune cells within the spheroid, as it leads to different nutrient levels and oxygen distribution across the structure. The unique morphology of C33A spheroids provides an opportunity to examine variations in tumor structure and treatment response, making them valuable for comparative studies with other, more compact spheroids, such as those formed by SiHa or HeLa cells [16,17].

Within the physiological context and dynamics of the 3D space available, a cancer tissue requires an unknown degree of flexibility and robustness for development. Adhesion and maturation of cells, and their underlying molecular mechanisms, have been extensively studied in cells grown as monolayers. During spheroid formation, cadherins or integrins bind to molecules that connect cells to their surroundings.

Recently, many cancer types, including breast cancer, CRC, and glioblastoma, have been subjected to mass spectrometry-based comparative quantitative profiling of cellular proteomes using cells grown in 2D and 3D cultures [18,19,20]. Previous studies have revealed noticeable differences in the proteomes of cells grown in 2D and 3D cultures. For instance, using proteome profiling of 2D- and 3D-cultured CRC cells, such as HT29, Yue et al. showed upregulation of proteins involved in energy metabolism and down regulation of those related to cell proliferation in 3D spheroids [21]. These findings suggest that 3D culture of cells can induce significant changes in their metabolic and proliferative profiles, which may have important implications for our understanding of cell behavior and its applications in various fields [22,23,24]. In a study evaluating the effect of three anticancer drugs cisplatin, resveratrol, and tirapazamine, as well as their combinations, on 2D and 3D cultures of human hepatocellular carcinoma cell line, HepG2, the size of the 3D spheroids was found to play a critical role in drug response [22]. This highlights the advantages of integrating 3D culture models into drug-screening applications. Furthermore, there is a growing trend of using in vitro cultures as “patient avatars” for developing personalized cancer treatments. In a study, chemotherapeutic drug response was examined in patient-derived xenograft models of high-grade ovarian cancer spheroids using a microfluidic platform [24].

**Figure 1:**
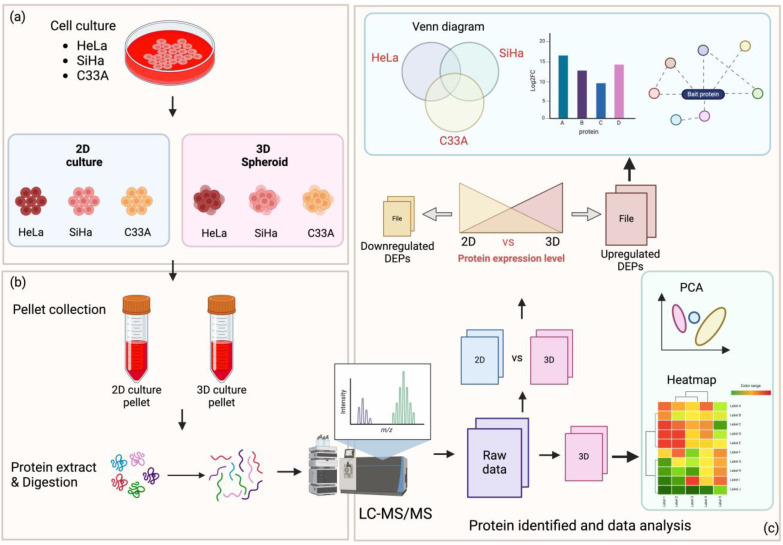
Proteomic workflow for 2D and 3D cervical cancer (CC) cell cultures. (**a**) CC cell lines, HeLa, SiHa, and C33A, were cultured and spheroids were formed. (**b**) Proteins were extracted from 2D and spheroid cell pellets. (**c**) LC-MS/MS showed a chromatogram representing the mass-to-charge ratio (*m*/*z*) of peptide intensity and the identification of peptides based on their fragmentation patterns. Data analysis was performed for six groups of samples. Proteins identified in 2D cell groups—2D-HeLa, 2D-SiHa, and 2D-C33A—were compared with their corresponding 3D cell groups. Protein levels within each cell type group were analyzed. Differentially expressed proteins (DEPs) indicated as “upregulated” showed higher levels in the 3D groups compared with those in the 2D groups. Upregulated and downregulated DEPs were further analyzed.

Despite the progress in research on spheroids, the relationship between various CC spheroid phenotypes and proteome levels remains unclear. Therefore, we investigated the mechanisms underlying the different phenotypes of CC cells using proteomic analysis employing liquid chromatography-mass spectrometry. The workflow for this study is illustrated in Figure 1.

## 2. Materials and Methods

### 2.1. Cervical Cancer Cell Culture

The CC cell lines used in this study were HeLa (an HPV-18-infected adenocarcinoma epithelial cell line, ATCC CCL-2) and SiHa (an HPV-16-infected squamous cell carcinoma cell line, ATCC HTB-35). HeLa and SiHa cell lines were purchased from the American Type Culture Collection (ATCC) (Manassas, VA, USA). C33A (an HPV-negative squamous cell carcinoma cell line, ATCC HTB-31). All the cell lines were maintained in Dulbecco’s modified Eagle medium, supplemented with 10% fetal bovine serum and 1% penicillin/streptomycin (Invitrogen, Berlin, Germany), at 37 °C under 5% CO_2_ atmosphere.

### 2.2. Preparation of Poly-HEMA-Coated Plates and Spheroid Formation

A poly-(2-hydroxyethyl methacrylate) (poly-HEMA; Sigma-Aldrich, St. Louis, MO, USA) solution was prepared at 50 mg/mL in ethanol by stirring with a magnetic bar for 8 h at room temperature. Then, 15 µL of this solution was added to each well of a 96-well U-bottom plate, and the plate was dried in an incubator for 4 h. For spheroid formation, 150 μL of complete medium containing 5000 cells was seeded in each well of the coated 96-well plate. The plate was incubated in a humidified incubator at 37 °C under 5% CO_2_ atmosphere. HeLa cells were incubated for three days to form spheroids, whereas SiHa and C33A cells were incubated for seven days. The medium was replenished every 72 h during the process of spheroid formation. Phase-contrast images of the spheroids were captured after their formation using LionheartFX live cell imaging (Biotek, Winooski, VA, USA).

### 2.3. Protein Extraction and Liquid Chromatography with Tandem Mass Spectrometry (LC-MS/MS) Sample Preparation

Cells were washed with phosphate-buffered saline to remove any contaminants. The washed cells were lysed in 1X reducing buffer using an ultrasonicator to extract proteins. The 4X reducing buffer contained 0.25 M Tris-HCl, 8% (*w*/*v*) SDS, 40% (*v*/*v*) glycerol, and 8% (*v*/*v*) β-mercaptoethanol. Protein concentration in the lysate was determined using the Bradford assay. A 20 μg aliquot of each sample was loaded on a sodium dodecyl sulfate-polyacrylamide gel, and all samples were electrophoresed together in a single batch.

Following electrophoresis, the gel bands corresponding to each sample were excised and cut into 2 × 2 mm segments. For in-gel trypsin digestion, these gel segments were first washed with deionized water. The staining dye was removed by incubating the segments in a 50% acetonitrile/50 mM ammonium bicarbonate solution. The proteins in the destained gel segments were reduced with 10 mM dithiothreitol (Sigma) and alkylated with 55 mM iodoacetamide (Sigma). The reduced proteins were subsequently digested with trypsin and Lys-C (Promega, Fitchburg, WI, USA) at an enzyme-to-substrate ratio of 1:50 for 20 h at 37 °C with gentle agitation. The digestion reaction was stopped by adding 5% formic acid/acetonitrile (1:2), and the peptides were separated from the gel by high-speed vertexing for 5 min. The resulting peptides were purified and desalted using C18 Solid Phase Extraction (SPE) disks (EMPORE™-3M, Saint Paul, MN, USA) and C18 beads, according to the STAGE tips protocol [1]. After purification, the peptides were dried using a vacuum centrifugal concentrator (LabConco, Kansas City, MO, USA) and resuspended in 0.1% formic acid to a final volume of 20 µL, yielding a concentration of 1 µg/µL. These peptide solutions were then used for LC-MS/MS analysis.

### 2.4. LC-MS/MS Analysis

Desalted peptide samples (2 µg) were injected into an ultra-high performance nanoflow LC system (Eksigent, Dublin, CA, USA) equipped with a C18 trap column (Nano Trap TP-1, 10 mm × 0.075 mm, 3 µm particle size, 120 Å pore size, Phenomenex, Torrance, CA, USA) and an analytical column (bioZen Peptide Polar C18 nanocolumn, 75 μm × 15 cm, 3 μm particle size, 120 Å pore size, Phenomenex). Gradient elution was performed using 0.1% formic acid in water as solvent A and 0.1% formic acid in acetonitrile as solvent B over 105 min. The *m*/*z* data were acquired using a TripleTOF 6600+ (ABSciex, Toronto, Canada) operated in positive ion mode on Sequential Window Acquisition of all Theoretical Mass Spectra (SWATH-MS). An information-dependent data acquisition (IDA) scan was performed at 100 ppm precursor mass tolerance and 0.2 Da fragment mass tolerance over a detection range of 300–1800 Da. The data-independent acquisition (DIA) mode was operated in the 7 and 1 *m*/*z* overlapping window. All *.wiff* data were subjected to protein identification and quantification using the Protein Pilot v.5.0.2.0 software (ABSciex) against the Swiss-Prot database (UniProtKB 2022_01) for *Homo sapiens* (20,385 proteins in the database). The search was performed using the following parameters: *IDA* carbaminomethyl (C) fixed modification, trypsin/Lys-C digestion, one missed cleavage, monoisotopic mass, and <0.01 false discovery rate (FDR); *DIA* 10 min extraction windows, 25 peptides/protein, 6 transitions/peptide, excluding shared peptides, 20 ppm extracted ion current width, and <0.01 FDR.

### 2.5. Bioinformatic and Statistical Analyses

Protein intensity was quantile-normalized and log2 transformed. The differentially expressed proteins (DEPs) between spheroid types were analyzed with an empirical Bayes moderated *t*-test using the *limma* package [2]. For 2D and 3D cultures of each cell type, the differential data with log_2_FC of 1 and *p*-value < 0.05 were considered significant and used for further analysis.

MetaboAnalyst 6.0 (https://www.metaboanalyst.ca/) was used to generate a heatmap of protein expression and to perform principal component analysis (PCA). PANTHER, which provides comprehensive information on the evolution of protein-coding gene families, was used for molecular function pathway analysis of data filtered based on the log_2_FC > 1 criterion. DEP datasets were analyzed for protein–protein interactions (PPI) using the STRING database (Version 12.0). cytoHubba was used to identify the hub proteins among the upregulated DEPs involved in the PPI network, based on the top 20 nodes ranked using the Maximal Clique Centrality method, and retrieved for functional enrichment using stringApp in the Cytoscape plugin program. The filtered data were used to perform gene ontology (GO) molecular function and cellular component enrichment analyses using the ShinyGO v0.77 version. All pathway analysis was performed using false discovery rate < 0.05. Statistical analyses were performed using the GraphPad Prism Software (version 9.00 for 141 macOS; GraphPad, San Diego, CA, USA).

### 2.6. Protein Validation by Western Blot Analysis

Spheroid pellets were lysed in 1X RIPA buffer (Pierce Biotechnology, Rockford, IL, USA). The lysate was mixed and incubated on ice for 20 min, followed by centrifugation at 12,000× *g*. Protein concentration was determined using the Bradford assay (Bio-Rad, Hercules, CA, USA). A total of 20 µg of protein extract was loaded onto a 12% SDS-PAGE gel, which was then transferred to polyvinylidene difluoride (PVDF) membranes (Amersham Pharmacia Biotech, Piscataway, NJ, USA). The membranes were blocked in 5% non-fat milk in Tris-buffered saline with 0.1% (*v*/*v*) Tween-20 (TBS-T) for 1 h at room temperature. Following blocking, the membranes were washed twice with TBS-T for 10 min.

Each membrane was incubated with primary antibodies (1:1000 dilution in 1% non-fat milk in TBS-T) targeting E-cadherin (cat. no. 24E10; 1:1000; Cell Signaling Technology, Inc., Danvers, MA, USA) and GAPDH (cat. no. 14C10; 1:1000; Cell Signaling Technology, Inc.), used as an internal control, overnight at 4 °C. After primary antibody incubation, the membranes were washed three times with TBS-T for 10 min each and then incubated with a secondary antibody (Anti-rabbit IgG horseradish peroxidase; cat. no. 7074; Cell Signaling Technology, Inc.) at a 1:2000 dilution in 1% non-fat milk in TBS-T for 2 h. The membranes were then washed again three times with TBS-T for 10 min.

Protein expression was visualized using an enhanced chemiluminescence reagent (Pierce™ ECL Western blotting Substrate, Thermo Fisher Scientific, Waltham, MA, USA). Densitometry was performed using a Chemiluminescence and Epi Fluorescence Alliance Q9 Advanced (Uvitech, Cambridge, UK) imager.

## 3. Results

Cancer research relies heavily on conventional 2D cell culture models. Nevertheless, this 2D approach fails to simulate the natural 3D cell environment, resulting in an inaccurate representation of the functional traits observed in living tissues. As such, spheroids represent a superior cell culture system for mimicking the in vivo conditions [25,26].

### 3.1. Generation and Characterization of HeLa, SiHa, and C33A Spheroids

**Figure 2:**
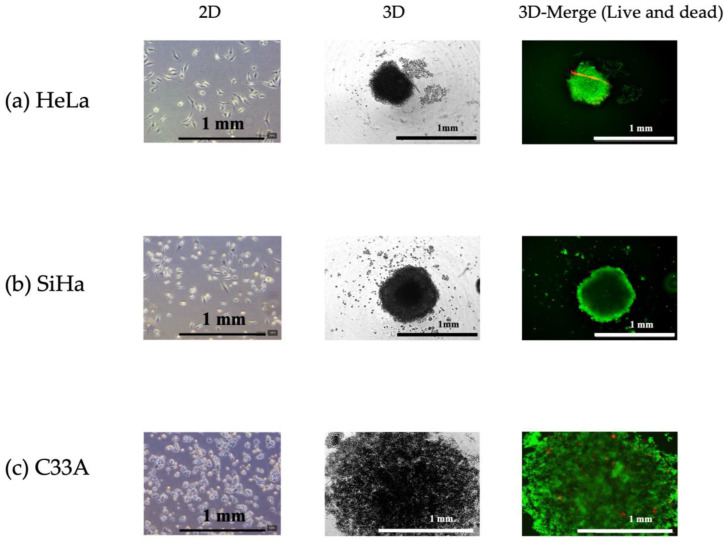
Morphology of cervical cancer (CC) spheroids and their characteristics. Phase contrast images at various passages of 2D cultures of (**a**) HeLa, (**b**) SiHa, and (**c**) C33A cells. Spheroids were formed by seeding 5000 cells/well in in-house-coated poly-(2-hydroxyethyl methacrylate) plates and incubating for 3 (for HeLa) or 7 (for SiHa and C33A) days. Spheroids were subjected to LIVE/DEAD staining (live = green; dead = red) and imaged using a LionheartFX live cell imager (×4 magnification).

The spheroids were formed as described in Section 2. After their formation, the viability of the spheroids was evaluated using a LIVE/DEAD staining kit. This kit employs two dyes—calcein AM (green fluorescence) to stain intracellular esterases, indicating live cells, and BOBO-3 iodide (red fluorescence) to assess membrane integrity, indicating dead cells. HeLa cells formed dense spheroids within 3 days (Figure 2a) unlike SiHa cells, which produced flatter spheroids. The SiHa spheroids had a flying saucer shape with a round and thick central region, whereas their periphery appeared flat (Figure 2b). SiHa and C33A cells were incubated for 7 days to achieve the formation of tight spheroids. Although SiHa cells did not form compact spheroids, the flat spheroids appeared more packed than the structure formed by C33A, which was more dispersed and loosely aggregated. In summary, HeLa and SiHa cells formed well-defined spheroids (Figure 2a,b), whereas C33A cells exhibited a more dispersed and loosely aggregated morphology (Figure 2c). Additionally, the LIVE/DEAD staining assay showed that after 3 (for HeLa cells) and 7 (for SiHa and C33A cells) days, spheroid formation was unaffected, and no dead cells were observed in the core of spheroids.

### 3.2. Proteomic Profiling and Analysis of DEPs in CC Spheroids

LC-MS/MS was used to assess the protein profiles of the CC spheroids. The spheroids showed different molecular characteristics. The raw LC-MS/MS data were grouped into 2D and 3D groups (Figure 1c). A thousand proteins were identified in both 2D and 3D of all cell types (Supplementary Table S1).

**Figure 3:**
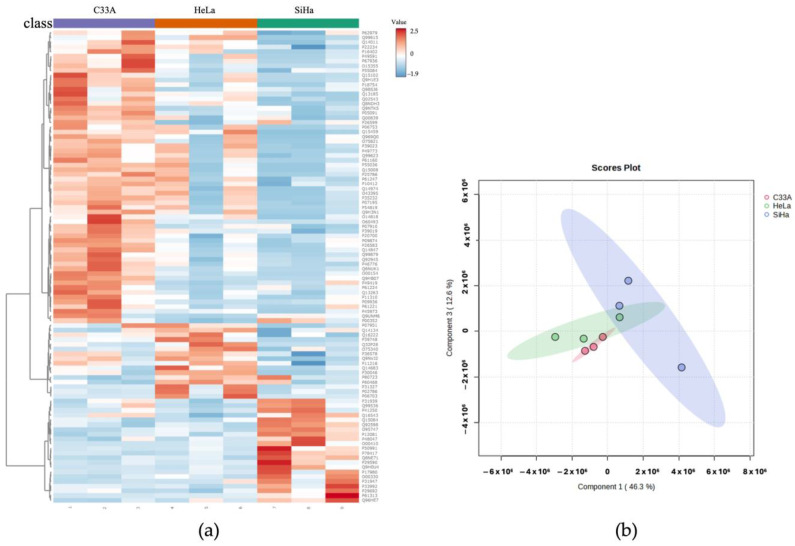
Overview of protein profiles in cervical cancer spheroids. (**a**) Heatmap of top 100 differentially expressed proteins in HeLa, SiHa, and C33A spheroids detected using mass spectrometry-based proteomics. The red and blue colors indicate upregulated and downregulated proteins in each type of spheroid. (**b**) Principal component analysis of overall protein profiles in the 3D-HeLa, 3D-SiHa, and 3D-C33A groups. Each green, blue and red dot represents the protein profiles of HeLa, SiHa and C33A spheroids, respectively. The colored circles represent 95% confidence intervals between different groups.

A heatmap was used to visualize the top 100 proteins with high expression levels in the 3D spheroids by comparing the CC cell groups (Figure 3a). Additionally, a PCA plot was generated to cluster the groups of proteins based on their expression profiles. The proteins in the HeLa cluster overlapped with those in the SiHa and C33A groups, whereas the SiHa and C33A groups formed distinct clusters based on their proteome profiles (Figure 3b).

**Figure 4:**
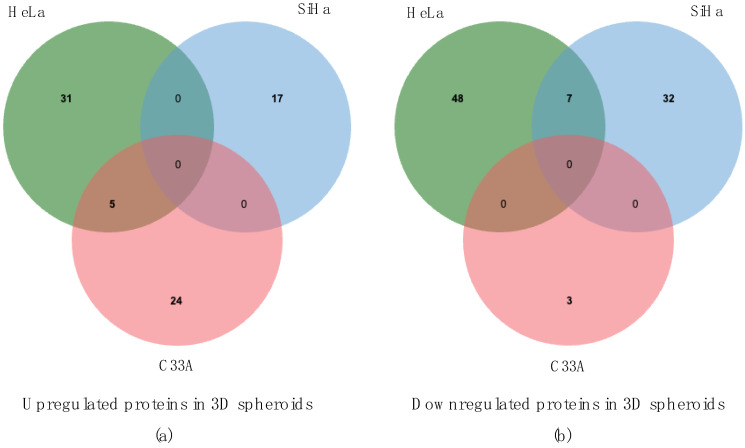
Profiles of differential expression proteins based on mass spectrometry results. Shared and unique proteins upregulated (**a**) and downregulated (**b**) in HeLa, SiHa, and C33A spheroids compared with that in respective 2D cultures.

To gain a clearer understanding of the dynamic changes in protein profiles between 2D cultures with adherent morphologies and 3D cultures with distinct morphologies, we conducted differential expression analysis on the proteome data. The DEPs were categorized into two groups: upregulated DEPs, which showed higher levels in the 3D group than in the 2D group, and downregulated DEPs, which showed higher levels in the 2D group than in the 3D group. After processing the DEP data, a Venn diagram was created (Figure 4).

The upregulated proteins in the 3D groups with log_2_fold change (FC) >1 and *p*-value < 0.05 are listed in Table 1, Table 2 and Table 3. The DEPs in each group differed, and they could be involved in determining the spheroid shapes. The HeLa group included proteins associated with binding activity, such as cadherin binding, cell adhesion molecule, and skeleton protein binding. Six of the 36 upregulated proteins in the 3D-HeLa group, including myosin heavy chain 9 (MYH9), vinculin (VCL), annexin A2 (ANXA2), chloride intercellular channel 1 (CLIC1), receptor for activated kinase 1 (RACK1), and Obg Like ATPase 1 (OLA1) have cadherin-binding activities. Additionally, 8 out of the 36 proteins, including MYH9, VCL, ANXA2, CLIC1, RACK1, ribosomal protein SA (RPSA), and OLA1, function in cell adhesion molecule binding. Notably, 9 of the 36 proteins, including MYH9, cofilin 1 (CFL1), VCL, torsin 1A interacting protein 1 (TOR1AIP1), ANXA2, adenylyl cyclase-associated protein 1 (CAP1), actinin (ACTN), cysteine and glycine-rich protein 1 (CSRP1), and calmodulin 3 (CALM3) are related to cytoskeleton binding. The Venn diagram shows the unique and shared proteins within each group. Figure 4a,b shows proteins with significantly different expression; the left panel highlights upregulated proteins in 3D spheroids, whereas the right panel shows downregulated proteins in each group. Figure 4a depicts the upregulated proteins in each 3D group, where 24 proteins were uniquely expressed in the loosely aggregated C33A spheroids, and 17 and 31 proteins were uniquely upregulated in the flat SiHa spheroids and compact HeLa spheroids, respectively. The upregulated proteins shared between the 3D-HeLa and 3D-C33A cells were BMS1 ribosome biogenesis factor (BMS1), TOR1AIP1, MYB binding protein 1a (MYBBP1A), proteasome 26S subunit, ATPase 4 (PSMC4), and X-ray repair cross-complementing 5 (XRCC5). The downregulated proteins in the 3D group are also plotted. As shown in Figure 4b, 3, 32, and 48 proteins were uniquely expressed in C33A, SiHa, and HeLa cells, respectively, showing a decreasing trend in ultralow attachment cultures. These uniquely expressed proteins may be responsible for features of spheroid formation, whereas the shared proteins may be linked to CC.

### 3.3. Molecular Function Analysis of Upregulated Proteins in 3D Spheroids Using the PANTHER Database

**Figure 5:**
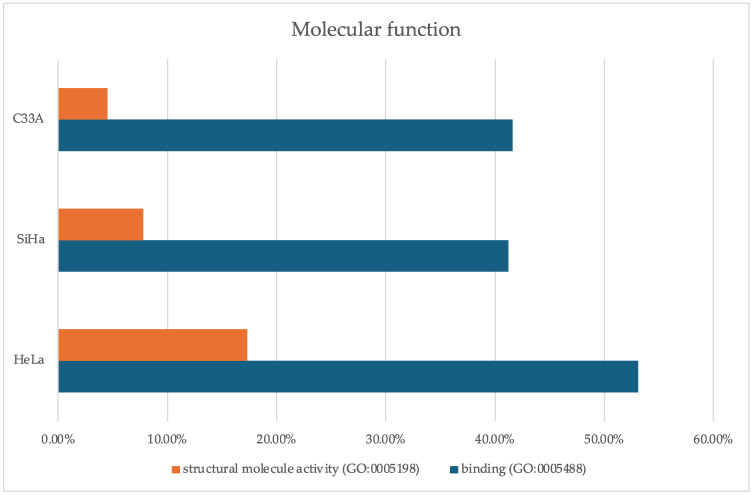
Upregulated proteins enriched for molecular functions terms in HeLa, SiHa, and C33A spheroids using the PANTHER database.

Data analysis was conducted to identify proteins that were upregulated and downregulated compared with their expression in the 2D groups. Molecular function analysis was performed using the PANTHER database. The results indicated that structural molecule activity and binding function terms were enriched by 53.10% and 17.30%, respectively, for the proteins upregulated in HeLa spheroids. In SiHa spheroids, structural molecule activity decreased to 41.20% compared with that in HeLa spheroids, and the binding activity was also lower than that in HeLa spheroids (Figure 5, Table 4). Similarly, in C33A spheroids, the upregulated proteins exhibited lower levels of terms related to structural molecule and binding activities than those in HeLa spheroids (Figure 5 and Table 4).

### 3.4. STRING Enrichment Network of Upregulated and Downregulated Proteins in 3D-CC Spheroids

**Figure 6:**
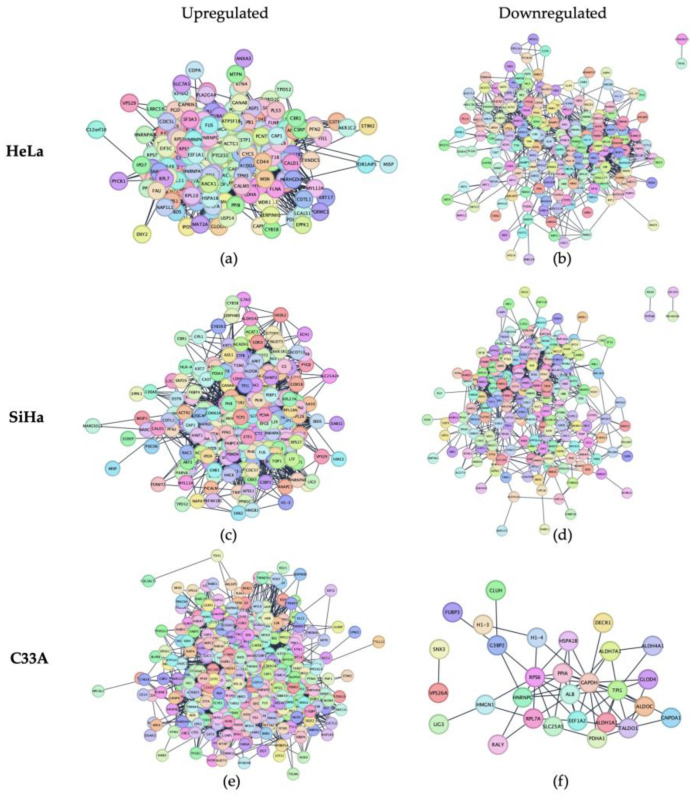
Protein–protein interaction networks of upregulated proteins of HeLa (**a**), SiHa (**c**) and C33A (**e**), and downregulated proteins of HeLa (**b**), SiHa (**d**) and C33A (**f**) spheroids determined using the STRING database.

The upregulated and downregulated proteins with a log2 FC cut-off of one were further evaluated for PPI using the STRING network, and protein clusters were identified using the Cytoscape plugin. Data imported into Cytoscape were retrieved from the STRING database. The PPI results are presented in Figure 6. The PPI networks for upregulated (Figure 6a,c,e) and downregulated (Figure 6b,d,f) proteins in each cell type are shown. Functional annotation of the upregulated proteins in all the groups was performed against the GO database using Cytoscape. Table 5 lists the molecular functions of cadherin binding, cell adhesion molecule binding, and binding activity for the upregulated proteins in the 3D groups. The upregulated proteins in HeLa spheroids showed an overrepresentation of molecular function-related binding activity. This suggests that the proteins identified among the upregulated proteins in the HeLa group are likely associated with molecular functions, making these findings reliable.

### 3.5. Identification of PPI Networks, Hub Proteins Among Upregulated Proteins in 3D CC Spheroids

**Figure 7:**
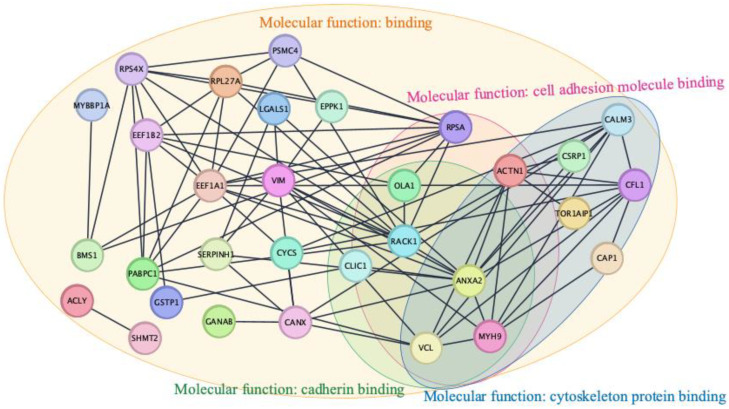
Protein–protein interaction networks of 36 significantly upregulated proteins in the 3D-HeLa spheroid group. Of the 36 proteins, 31 are related with molecular function of binding, cadherin binding, and cell adhesion molecule binding.

To identify the PPI in each group of samples, the STRING network with upregulated proteins exhibiting log_2_FC > 1 and *p*-value < 0.05, was used to identify the critical hub proteins among the top ten identified proteins and to retrieve their functions using the STRING database. Additionally, the PPIs of the filtered upregulated proteins in 3D-HeLa spheroids, as depicted in Figure 7, were constructed using the Cytoscape plug-in STRING apps. Ten hub proteins were categorized based on the PPI network (Figure S1). The upregulated hub proteins among the common DEPs were ranked based on their degree of connectivity with other proteins. The pseudocolor scale from red to yellow represents a protein ranking from one to ten. Dark red, orange, and yellow represent the highest, intermediate, and lowest degrees, respectively. As shown in Figure 7, most of the upregulated hub proteins in 3D-HeLa spheroids were involved in cell binding, cell adhesion molecule, cadherin binding, and cytoskeleton protein binding. Notably, no relationship between cell junction and cadherin binding was found for the significantly upregulated proteins in 3D-SiHa and 3D-C33A cells (Figures S2 and S3).

### 3.6. Modulation of Cadherin-Associated, Cytoskeleton, and Focal Adhesion Proteins in 3D CC Spheroids Compared with That in Monolayer Culture Cells

**Figure 8:**
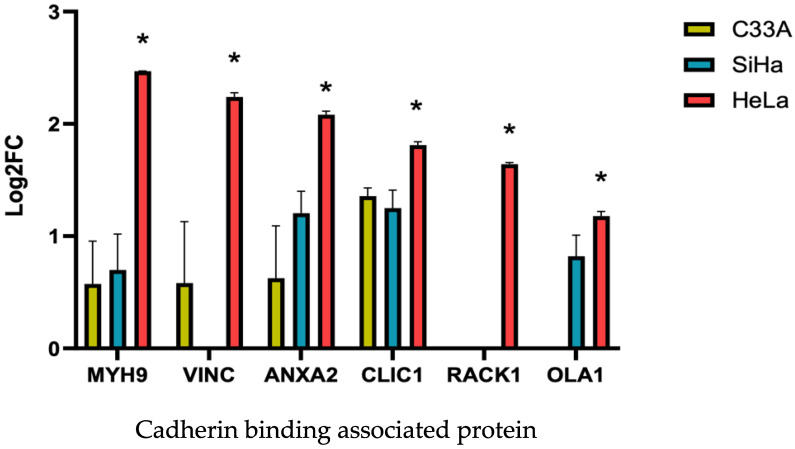
The log_2_FC values of cadherin binding associated protein. Significant differences between 3D spheroids and 2D-cultured cells for HeLa, SiHa, and C33A are indicated with an asterisk (* *p* < 0.05).

In the context of spheroid formation, we complemented the understanding of adhesion in spheroid formation by evaluating the identified proteins related to cadherin adhesion, cytoskeleton, such as actin and microtubules, and focal adhesion kinase (FAK). MYH9, VINC, ANXA2, CLIC1, RACK1, and OLA1 are cadherin binding-associated proteins. Comparing 3D and 2D cultures, all six proteins were significantly upregulated in compact HeLa spheroids compared with those in monolayer cultures (Figure 8, Table 1). In SiHa and C33A cells, none of the six proteins showed significant differences between 3D and 2D cultures (Figure 8). The proteins are depicted in Table 1. Thirty-six proteins were significantly upregulated in 3D-HeLa cells and exhibited binding activity. Twenty of the 36 proteins were associated with cell adhesion and cadherin binding. MYH9, VINC, ANXA2, CLIC1, RACK1, and OLA1 are not only associated with cadherin binding, but also show enriched molecular functions of binding activity, cell adhesion molecule binding, and cytoskeleton binding activity (Table 6). Importantly, we investigated whether focal adhesion and cell junction proteins enriched in the cellular component pathways impact cellular processes, such as cell–cell adhesion. We found that some upregulated proteins in HeLa spheroids, such as VIME, MYH9, COF1, VINC, CAP1, ACTN1, PABP1, RS4X, and CSRP1, were enriched in the cellular component terms of focal adhesion and cell junction (Table 6).

### 3.7. GO Analysis and Signaling Pathway Enrichment of Upregulated Proteins in 3D CC Spheroids

For assessing protein datasets, GO offers a descriptive background as well as functional annotation and classification. We filtered the upregulated proteins with log_2_FC > 1 and *p*-value < 0.05 and further analyzed them using ShinyGO v0.80 (https://bioinformatics.sdstate.edu/go (accessed on 3 September 2024)), a GO tool. Most molecular functions associated with the upregulated proteins in 3D HeLa spheroids were related to binding activities, such as intermediate filament binding, ribosome binding, actin filament binding, cadherin binding, cell adhesion molecule binding, actin binding, cytoskeleton protein binding, and protein-containing complex binding, which were enriched in DEPs (Figure S4a). Cellular components showed that the proteins upregulated in 3D-HeLa cells were primarily linked to the actin cytoskeleton, cell–substrate junction, focal adhesion, cell leading edge, anchoring junction, and cell junction (Figure S4a). The molecular function and cellular component terms related to the upregulated proteins in the 3D-SiHa and 3D-C33A cells were also investigated. The molecular function terms for 3D-SiHa were related to DNA and RNA binding levels (Figure S4b). The cellular components related to the upregulated proteins in 3D-SiHa were enriched in cytosolic large ribosome, cortical cytoskeleton, actin filament, vesicle lumen, extracellular vesicle, mitochondrion, and vesicle (Figure S4b). Additionally, the molecular functions enriched in C33A were strongly associated with snoRNA, tau protein, antioxidant activity, purine nucleotide binding, and carbohydrate binding (Figure S4c). As depicted in Figure S5c, the largest fraction of upregulated proteins in 3D-C33A cells was associated with the cellular component function of the protein kinase 5 complex, translation release factor complex, and Ku70: Ku80 complex.

**Figure 9:**
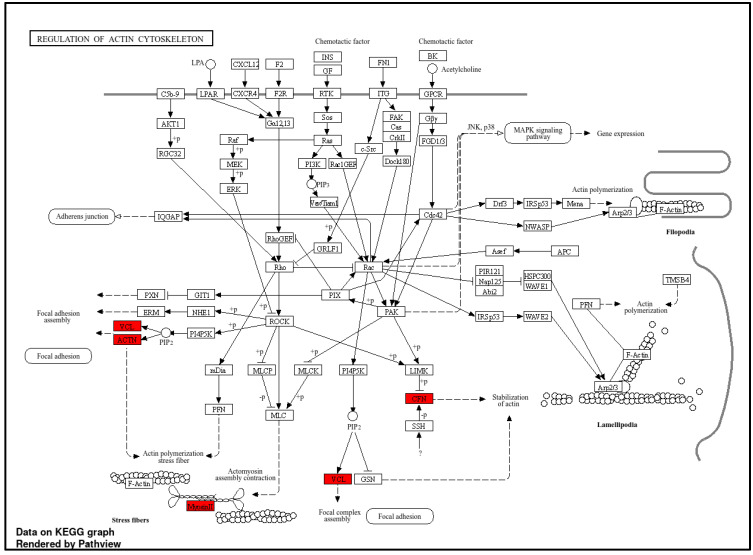
Gene ontology (GO) Kyoto encyclopedia of genes and genomes (KEGG) analysis of regulation of actin cytoskeleton terms for upregulated proteins in HeLa spheroids using ShinyGO.

Among the upregulated proteins in HeLa spheroids, we identified 36 significantly upregulated and 31 unique proteins (Figure 3a). Pathway analysis showed that the proteins upregulated in HeLa spheroids were mainly enriched in ribosome and regulation of actin cytoskeleton signaling pathway (Figure 9). Notably, among the upregulated proteins in 3D-SiHa and 3D-C33A cells, neither binding nor cytoskeleton signaling pathways were found.

### 3.8. E-Cadherin Protein Validation in CC Spheroids

**Figure 10:**
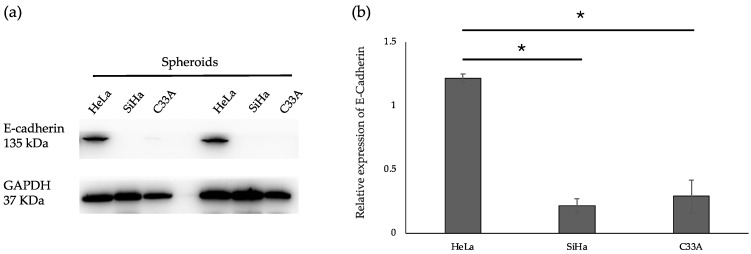
The quantitative analysis of E-cadherin protein expressed shows differences between various type of spheroids. Western blot analysis was performed with protein extracts from spheroids culture. Spheroids were lysed in RIPA buffer and analyzed by SDS-PAGE. Antibodies against E-cadherin was used. GAPDH is shown as internal control (**a**). E-cadherin was normalized to GAPDH band intensity (**b**). Data are shown as the mean ± SD of quadruplicate experiments. Statistical analysis was performed using Student’s *t*-test. Significance: * *p*-value < 0.05 compared between group.

Our proteomic data revealed that cadherin-associated proteins were highly expressed in HeLa spheroids compared to HeLa conventional cultures and spheroids of SiHa and C33A. Based on these findings, we analyzed protein extracts from CC spheroids to detect E-cadherin. We observed variations in E-cadherin protein levels among the different CC spheroids, with HeLa spheroids showing significantly stronger E-cadherin expression compared to the other cell lines (Figure 10). This suggests that differences in E-cadherin expression may be linked to adhesion processes that play a role in spheroid formation.

## 4. Discussion

In this study, we used poly-HEMA-coated plates to form CC spheroids from CC cell lines, including HeLa (HPV16 infected), SiHa (HPV18 infected), and C33A (uninfected). The poly-HEMA-coated plate is essential for the spheroid generation to prevent cells from attaching to the culture substrate, thereby enhancing cell–cell adhesion and resulting in a well-defined spherical structure. The formation of spheroids in conventional cultures of CC cell lines has been reported. We found that HeLa and SiHa cells formed compact and flat spheroids, respectively. However, C33A exhibited a loosely aggregated structure. It has been hypothesized that HPV proteins may contribute to spheroid formation. Muniandy et al. revealed that the E7 oncoprotein derived from HPV does not correlate with spheroid formation in non-HPV-infected C33A cells [13]. They incorporated the HPV-E7 oncoprotein into C33A cells; however, C33A cells could not form compact or flat spheroids, similar to the HeLa and SiHa cell lines [13]. We found that the HeLa, SiHa, and C33A cell lines exhibited consistent and highly reproducible forms across wells and plates and showed variability in the shapes of the spheroids.

Previous studies successfully identified the mechanism of spheroid formation. Spheroid formation involves at least three steps: (i) initial aggregation, (ii) cell compaction, and (iii) spheroid growth. However, phase transitions do not occur promptly. The characterization of each phase indicates that a certain process dominates the phase [27]. Smyrek et al. investigated the role of different proteins in various breast cancer cell lines, including cadherin, actin, microtubule network, and FAK, and hypothesized that they have an adhesion function during spheroid formation [27].

The involvement of cadherins and integrins, which connect the cells to their environment, is the main focus in spheroid formation [27,28]. Few studies have investigated the relationship between actin and microtubules during spheroid formation. Actin and microtubules are involved in the adhesion processes in conventional cultures [29,30,31,32,33]. Moreover, FAK is also essential for cell adhesion, growth, and migration [34,35,36]. During spheroid formation, a small proportion of different cancer cell lines, such as colon cancer, cannot integrate into spheroids, and the loss of cell–cell adhesion molecules, such as E-cadherin, catenin, and P-cadherin, in SW20, DLD-20, and HCT116 cell lines is responsible for non-spheroid-forming phenotypes [28]. We performed proteomic analysis to determine whether the same cancer cell types formed different spheroid structures having different shapes. Several mechanisms contribute to the formation of these different structures. We investigated the correlation between upregulated proteins in HeLa spheroids and their compact shape structures. The proteins that were significantly upregulated in compact HeLa spheroids were not expressed in other cells. Among the 36 significantly upregulated proteins in HeLa spheroids, 20 were related to binding activity and 6 (MYH9, VCL, ANXA2, CLIC1, RACK, and OLA1) were enriched in cadherin-binding function. Smyrek et al. suggested that cadherin is indispensable for spheroid formation. They examined whether cadherin is imperative for spheroid formation. They found that after inhibition of cadherin function with the DECMA-1 antibody, none of the various breast cancer cell lines, T47D (infiltrating ductal carcinoma of breast cancer), 4T1 (stage IV human breast cancer cells), and HC11 (mouse epithelial breast cells), were able to form spheroids [27]. This is consistent with the enrichment of cadherin-associated proteins in HeLa spheroids in our study and cadherins might be involved in the formation of compact HeLa spheroids [28]. Western blot analysis revealed that E-cadherin expression was significantly higher in HeLa spheroids compared to other cervical cancer (CC) spheroids (Figure 10). Cadherins, including E-cadherin, are critical mediators of cell–cell adhesion and play a pivotal role in spheroid formation. Previous studies have shown that blocking E-cadherin impairs spheroid formation, underscoring its importance in this process. However, E-cadherin’s role does not involve increasing the probability of cell contact during collisions. Instead, its primary function appears to be stabilizing existing cell–cell contacts rather than initiating new ones [30]. Furthermore, MYH9, a cadherin-associated protein, promotes the accumulation of E-cadherin and facilitates the formation of adherent junctions [37]. Taken together, these findings suggest that E-cadherin, in conjunction with cadherin-associated proteins such as MYH9, may collaborate to drive the initial steps of spheroid formation, stabilizing cell–cell interactions necessary for the structural integrity of spheroids. Actin cytoskeleton is also essential for spheroid formation. The actin cytoskeleton plays a role in the adhesion and regulation of cell shape. When cytoskeletal polymerization was blocked during the growth of actin filaments, the cells failed to form spheroids. Moreover, the cells exhibited no proper connections and were fragile during pipetting [27]. FAK is involved in the regulation of cell adhesion, proliferation, and movement. Its main role is to transmit extracellular signals via integrins and to affect cell adhesion and migration by reorganizing actin filaments and microtubules. FAK has also been investigated in mature spheroids [27,34]. We discovered that the upregulated proteins in HeLa spheroids were enriched in the molecular function of focal adhesion (Figure S4a). Interestingly, no focal adhesion function was observed in the upregulated SiHa and C33A 3D cultures (Figure S4b,c). Cell aggregation is mediated by cell–cell adhesion and can be influenced by cell–ECM adhesion.

**Figure 11:**
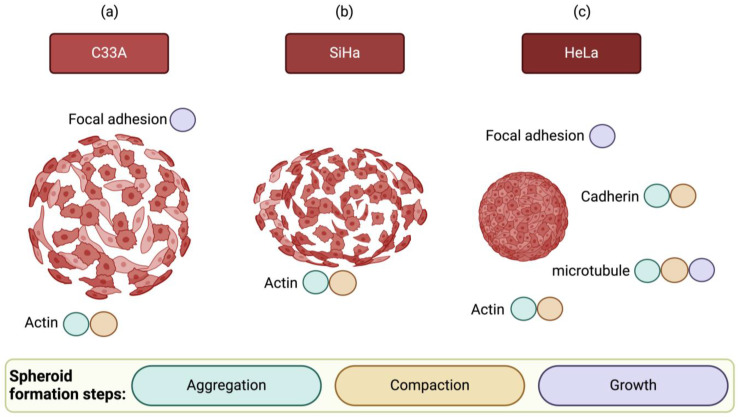
Model illustrating the phase-dependent roles of intracellular components in the cervical cancer 3D culture model for C33A (**a**), SiHa (**b**) and HeLa (**c**), highlighting the involvement of adhesion molecules at each stage. The colored circles indicate the key molecules essential for specific phases.

Using the liquid overlay method to form spheroids, researchers identified that three intercellular components, cadherin, actin, and microtubules, play dominant roles in each of the three phases of spheroid formation [27]. During the aggregation phase, cadherin and actin are predominant. Microtubules play a major role in the compaction phase by acting alongside cadherin and actin. Microtubules and FAK are key players in the subsequent growth phases [27]. Overall, in SiHa and C33A cells, none of the key proteins necessary for spheroid formation was affected to form compact spheroids (Figure 11a,b). These findings prove that HeLa cells can form a compact spheroid shape because during the remodeling process, they upregulate proteins essential for spheroid formation, as depicted in Figure 11c.

## 5. Conclusions

Proteomic analysis revealed the differences in protein levels related to the development of compact spheroids and loose aggregates. Among the three CC lines examined, HeLa cells formed compact spheroids because they express essential proteins required for all the stages of spheroid formation. Cadherin-related proteins and actin are key protein groups required during the aggregation phase. In compact HeLa spheroids, proteins, such as MYH9, VINC, ANXA2, CLIC, RACK1, OLA1, COF1, CAP1, ACTN1, CSRP1, and CALM3, function as adhesion molecules. In the second phase, which is the compaction phase, microtubule proteins, including TOR1AIP1, CAP1, CSRP1, MYH9, VINC, COF1, ANXA2, and CALM3, play a role in the rearrangement and maintenance of spheroids. Finally, during the proliferation or growth phase, microtubules and focal adhesion molecules, such as VIME, MYH9, COF1, VINC, CAP1, ACTN1, PABP1, RS4X, CSRP1, PRS6B, CALX, EPIPL, and CALM3, are crucial. SiHa and C33A cells did not form fully compact spheroids because they lack the essential proteins required at different stages of spheroid formation (Figure 11). Although the proteomic analysis provides valuable insights into the proteins associated with spheroid formation, a limitation of this study is that the sensitivity of mass spectrometry may not be sufficient to identify all proteins, particularly those present at low abundance. Mass spectrometry tends to favor the detection of more abundant proteins, while low-abundance proteins may be underrepresented. This could result in an incomplete proteomic profile and limit the discovery of potentially important proteins related to spheroid formation such as E-cadherin which was not detected in our analysis. Despite its absence in the proteomic data, E-cadherin was selected for validation due to its known interactions with cadherin-associated proteins, which are relevant to spheroid formation. However, in future studies, we aim to validate these findings, ideally via functional assays or complementary proteomic techniques, to confirm the roles of identified proteins and to ensure the robustness and applicability of our conclusions.

In conclusion, our findings suggest that a physiological understanding of CC cell adhesion and growth can be achieved using a dynamic 3D culture model. Our results highlight the key proteins involved in the formation of compact spheroids, particularly in CC cell lines.

## Figures and Tables

**Table 1:** Upregulated proteins in 3D-HeLa spheroids (vs. 2D cell culture) with log_2_FC > 1 and *p* < 0.05.

No.	Protein_Name	Protein_Id	logFC	*p*-Value
1	VIME	P08670	2.823	0.001
2	LEG1	P09382	2.542	0.008
3	BMS1	Q14692	2.490	0.023
4	MYH9	P35579	2.470	0.002
5	LIS1	P43034	2.285	0.049
6	COF1	P23528	2.262	0.028
7	VINC	P18206	2.239	0.039
8	TOIP1	Q5JTV8	2.215	0.020
9	GSTP1	P09211	2.200	0.003
10	MBB1A	Q9BQG0	2.130	0.038
11	ANXA2	P07355	2.082	0.031
12	MYG1	Q9HB07	2.019	0.045
13	4F2	P08195	1.994	0.028
14	EF1A1	P68104	1.975	0.037
15	PRS6B	P43686	1.963	0.007
16	CLIC1	O00299	1.812	0.029
17	CAP1	Q01518	1.807	0.022
18	EF1B	P24534	1.783	0.026
19	ACTN1	P12814	1.727	0.047
20	SERPH	P50454	1.726	0.035
21	CYC	P99999	1.665	0.017
22	PABP1	P11940	1.657	0.026
23	GANAB	Q14697	1.645	0.042
24	RACK1	P63244	1.641	0.015
25	RS4X	P62701	1.563	0.014
26	CSRP1	P21291	1.475	0.016
27	CALX	P27824	1.464	0.028
28	GLYM	P34897	1.439	0.047
29	RSSA	P08865	1.369	0.044
30	XRCC5	P13010	1.355	0.019
31	RL27A	P46776	1.350	0.045
32	EPIPL	P58107	1.326	0.024
33	CALM3	P0DP25	1.241	0.026
34	OLA1	Q9NTK5	1.178	0.041
35	TPD52	P55327	1.160	0.044
36	ACLY	P53396	1.139	0.035

**Table 2:** Upregulated proteins in 3D-SiHa spheroids (vs. 2D cell culture) with log_2_FC > 1 and *p* < 0.05.

No.	Protein_Name	Protein_Id	logFC	*p*-Value
1	COTL1	Q14019	2.684	0.007
2	CPSM	P31327	2.587	0.005
3	CISY	O75390	2.427	0.006
4	DOPD	P30046	2.275	0.041
5	C1TC	P11586	2.206	0.046
6	DNJA1	P31689	1.966	0.009
7	ANXA1	P04083	1.905	0.034
8	RL13	P26373	1.812	0.033
9	CBR1	P16152	1.692	0.019
10	TBB6	Q9BUF5	1.658	0.030
11	DEST	P60981	1.602	0.050
12	RL30	P62888	1.557	0.028
13	RS27A	P62979	1.505	0.028
14	ETFB	P38117	1.472	0.029
15	RIR1	P23921	1.431	0.018
16	PYGB	P11216	1.218	0.048
17	XRCC6	P12956	1.209	0.047

**Table 3:** Upregulated proteins in 3D-C33A spheroids (vs. 2D cell culture) with log_2_FC > 1 and *p* < 0.05.

No.	Protein_Name	Protein_Id	logFC	*p*-Value
1	SYYC	P54577	3.051	0.007
2	CDK5	Q00535	2.831	0.021
3	PTMA	P06454	2.730	0.042
4	MGST1	P10620	2.692	0.008
5	1433S	P31947	2.685	0.004
6	NQO1	P15559	2.567	0.016
7	PZP	P20742	2.380	0.037
8	BMS1	Q14692	2.247	0.038
9	TOIP1	Q5JTV8	2.144	0.018
10	RL15	P61313	2.087	0.004
11	AL3A1	P30838	2.084	0.014
12	MBB1A	Q9BQG0	1.995	0.041
13	CSN3	Q9UNS2	1.994	0.011
14	PRS6B	P43686	1.969	0.003
15	ACTB	P60709	1.880	0.022
16	MTAP	Q13126	1.824	0.029
17	SIAS	Q9NR45	1.762	0.013
18	ARPC3	O15145	1.734	0.041
19	RS7	P62081	1.674	0.044
20	SRRM2	Q9UQ35	1.569	0.026
21	VASP	P50552	1.541	0.025
22	ASSY	P00966	1.497	0.041
23	HS105	Q92598	1.265	0.046
24	ERF1	P62495	1.254	0.021
25	EHD2	Q9NZN4	1.225	0.050
26	GSHR	P00390	1.128	0.047
27	IF4A3	P38919	1.124	0.026
28	HARS1	P12081	1.073	0.042
29	XRCC5	P13010	1.053	0.047

**Table 4:** Percentage of upregulated proteins in spheroids involved in molecular function pathways as analyzed using the PANTHER database.

Description	HeLa	SiHa	C33A
	% Proteins with Molecular Function		
Binding (GO:0005488)	53.10	41.20	41.60
Structural molecule activity (GO:0005198)	17.30	7.8	4.5

**Table 5:** STRING enrichment table of upregulated proteins in 3D cell cultures.

Category	Description	HeLa		SiHa		C33A	
		FDR Value	*p*-Value	FDR Value	*p*-Value	FDR Value	*p*-Value
GO molecular function	Cadherin binding (MYH9, VINC, ANXA2, CLIC1, RACK, OLA1)	6.01 × 10^−34^	2.44 × 10^−37^	3.60 × 10^−20^	4.38× 10^−23^	1.42 × 10^−21^	1.44× 10^−24^
	Cell adhesion molecule binding (MYH9, VINC, ANXA2, CLIC1, ACTN1, RACK1, RSSA, OLA1)	3.34 × 10^−31^	2.03 × 10^−34^	3.88 × 10^−18^	5.50 × 10^−21^	7.99 × 10^−15^	1.46 × 10^−17^
	Binding (VIME, MYH9, LIS1, COF1, VINC, ACTN1, PABP1, RACK, RS4X, CALM3, OLA1, etc.)	1.94 × 10^−27^	2.75 × 10^−30^	1.18 × 10^−21^	1.20 × 10^−24^	3.34 × 10^−24^	2.71 × 10^−27^
GO cellular component	Focal adhesion (VIME, MYH9, COF1, VINC, CAP1, ACTN1, PABP1, RS4X, CSRP1)	1.68 × 10^−46^	8.21 × 10^−49^	1.34 × 10^−22^	1.05 × 10^−24^	1.77 × 10^−14^	1.81 × 10^−16^
	Cell junction (VIME, MYH9, COF1, VINC, ANXA2, PRS6B, CAP1, ACTN1, PABP1, RS4X, CSRP1, CALX, EPIPL, CALM3)	8.54 × 10^−29^	5.84 × 10^−31^	3.65 × 10^−12^	5.35 × 10^−14^	2.40 × 10^−10^	4.45 × 10^−12^

**Table 6:** List of 20 significantly upregulated HeLa proteins enriched in molecular function (binding) and cellular component (focal adhesion) terms.

No.	Molecular Function				Cellular Component	
	Binding Activity	Cell Adhesion Molecule Binding	Cadherin Binding	Cytoskeleton Protein Binding	Focal Adhesion	Cell Junction
1	VIME				VIME	VIME
2	MYH9	MYH9	MYH9	MYH9	MYH9	MYH9
3	LIS1					
4	COF1			COF1	COF1	COF1
5	VINC	VINC	VINC	VINC	VINC	VINC
6	ANXA2	ANXA2	ANXA2	ANXA2		ANXA2
7	PRS6B					PRS6B
8	CLIC1	CLIC1	CLIC1			
9	CAP1			CAP1	CAP1	CAP1
10	ACTN1	ACTN1		ACTN1	ACTN1	ACTN1
11	PABP1				PABP1	PABP1
12	GANAB					
13	RACK1	RACK1	RACK1			
14	RS4X				RS4X	RS4X
15	CSRP1			CSRP1	CSRP1	CSRP1
16	CALX					CALX
17	RSSA	RSSA				
18	EPIPL					EPIPL
19	CALM3			CALM3		CALM3
20	OLA1	OLA1	OLA1			

## Data Availability

The raw MS/MS data and analysis files have been deposited to the ProteomeXchange Consortium (https://proteomecentral.proteomexchange.org/ (accessed on 3 December 2024)) via the jPOST partner repository (https://jpostdb.org/) with the dataset identifier JPST003431 and PXD056969.

## Supplementary Materials

The following supporting information can be downloaded at: https://www.mdpi.com/article/10.3390/cells13232004/s1, Figure S1. Subnetwork of the top 10 hub proteins in the protein–protein interaction network of upregulated proteins in 3D-HeLa spheroids. The color of the node reflects the degree of connectivity. Figure S2. Protein–protein interaction networks of eight significantly upregulated proteins in the 3D-SiHa spheroid group. Figure S3. Protein–protein interaction networks of 29 significantly upregulated proteins in the 3D-C33A spheroid group. Figure S4. Gene ontology (GO) analysis of molecular function using the ShinyGO tool (v.0.77). Figure S5. Gene ontology (GO) analysis of cellular component using the ShinyGO tool (v.0.77). Figure S6. The histograms of normalized abundance values of all samples. Table S1. Total proteins identified from mass spectrometry. Table S2. Downregulated proteins in 3D-HeLa spheroids with log_2_FC < −1 and *p* < 0.05. Table S3. Downregulated proteins in 3D-SiHa spheroids with log_2_FC < −1 and *p* < 0.05. Table S4. Downregulated proteins in 3D-SiHa spheroids with log_2_FC < −1 and *p* < 0.05.

## References

[1] World Health Organization (2020). Cervical Cancer.

[2] Arora S., Singh S., Mittal A., Desai N., Khatri D.K., Gugulothu D., Lather V., Pandita D., Vora L.K. (2024). Spheroids in cancer research: Recent advances and opportunities. J. Drug Deliv. Sci. Technol..

[3] Nath S., Devi G.R. (2016). Three-dimensional culture systems in cancer research: Focus on tumor spheroid model. Pharmacol. Ther..

[4] Elliott N.T., Yuan F. (2011). A review of three-dimensional in vitro tissue models for drug discovery and transport studies. J. Pharm. Sci..

[5] Costa E.C., Moreira A.F., de Melo-Diogo D., Gaspar V.M., Carvalho M.P., Correia I.J. (2016). 3D tumor spheroids: An overview on the tools and techniques used for their analysis. Biotechnol. Adv..

[6] Hoarau-Véchot J., Rafii A., Touboul C., Pasquier J. (2018). Halfway between 2D and animal models: Are 3D cultures the ideal tool to study cancer-microenvironment interactions?. Int. J. Mol. Sci..

[7] Friedrich J., Seidel C., Ebner R., Kunz-Schughart L.A., Drug Screen S.B. (2009). Spheroid-based drug screen: Considerations and practical approach. Nat. Protoc..

[8] Antoni D., Burckel H., Josset E., Noel G. (2015). Three-dimensional cell culture: A breakthrough in vivo. Int. J. Mol. Sci..

[9] Shaheen S., Ahmed M., Lorenzi F., Nateri A.S. (2016). Spheroid-formation (colonosphere) assay for in vitro assessment and expansion of stem cells in colon cancer. Stem Cell Rev. Rep..

[10] Sodek K.L., Ringuette M.J., Brown T.J. (2009). Compact spheroid formation by ovarian cancer cells is associated with contractile behavior and an invasive phenotype. Int. J. Cancer.

[11] Ivascu A., Kubbies M. (2007). Diversity of cell-mediated adhesions in breast cancer spheroids. Int. J. Oncol..

[12] Sargenti A., Musmeci F., Bacchi F., Delprete C., Cristaldi D.A., Cannas F., Bonetti S., Pasqua S., Gazzola D., Costa D. (2020). Physical characterization of colorectal cancer spheroids and evaluation of NK cell infiltration through a flow-based analysis. Front. Immunol..

[13] Muniandy K., Asra Ahmad Z., Annabel Dass S., Shamsuddin S., Mohana Kumaran N., Balakrishnan V. (2021). Growth and invasion of 3D spheroid tumor of HeLa and CasKi cervical cancer cells. Oncologie.

[14] Minamikawa-Tachino R., Ogura K., Ito A., Nagayama K. (2020). Time-lapse imaging of HeLa spheroids in soft agar culture provides virtual inner proliferative activity. PLoS ONE.

[15] Geiger F., Schnitzler L.G., Brugger M.S., Westerhausen C., Engelke H. (2022). Directed invasion of cancer cell spheroids inside 3D collagen matrices oriented by microfluidic flow in experiment and simulation. PLoS ONE.

[16] Zhang J., Rashmi R., Inkman M., Jayachandran K., Ruiz F., Waters M.R., Grigsby P.W., Markovina S., Schwarz J.K. (2021). Integrating imaging and RNA-seq improves outcome prediction in cervical cancer. J. Clin. Investig..

[17] Kutle I., Polten R., Hachenberg J., Klapdor R., Morgan M., Schambach A. (2023). Tumor organoid and spheroid models for cervical cancer. Cancers.

[18] Yue X., Lukowski J.K., Weaver E.M., Skube S.B., Hummon A.B. (2016). Quantitative proteomic and phosphoproteomic comparison of 2D and 3D colon cancer cell culture models. J. Proteome Res..

[19] He W., Kuang Y., Xing X., Simpson R.J., Huang H., Yang T., Chen J., Yang L., Liu E., He W. (2014). Proteomic comparison of 3D and 2D glioma models reveals increased HLA-E expression in 3D models is associated with resistance to NK cell-mediated cytotoxicity. J. Proteome Res..

[20] Morrison B.J., Hastie M.L., Grewal Y.S., Bruce Z.C., Schmidt C., Reynolds B.A., Gorman J.J., Lopez J.A. (2012). Proteomic comparison of Mcf-7 tumoursphere and monolayer cultures. PLoS ONE.

[21] Molika P., Leetanaporn K., Rungkamoltip P., Roytrakul S., Hanprasertpong J., Navakanitworakul R. (2023). Proteomic analysis of small extracellular vesicles unique to cervical cancer. Transl. Cancer Res..

[22] Patra B., Peng C.C., Liao W.H., Lee C.H., Tung Y.C. (2016). Drug testing and flow cytometry analysis on a large number of uniform sized tumor spheroids using a microfluidic device. Sci. Rep..

[23] Fröhlich E. (2020). Issues with cancer spheroid models in therapeutic drug screening. Curr. Pharm. Des..

[24] Dadgar N., Gonzalez-Suarez A.M., Fattahi P., Hou X., Weroha J.S., Gaspar-Maia A., Stybayeva G., Revzin A. (2020). A microfluidic platform for cultivating ovarian cancer spheroids and testing their responses to chemotherapies. Microsyst. Nanoeng..

[25] Edmondson R., Broglie J.J., Adcock A.F., Yang L. (2014). Three-dimensional cell culture systems and their applications in drug discovery and cell-based biosensors. Assay Drug Dev. Technol..

[26] Stratmann A.T., Fecher D., Wangorsch G., Göttlich C., Walles T., Walles H., Dandekar T., Dandekar G., Nietzer S.L. (2014). Establishment of a human 3D lung cancer model based on a biological tissue matrix combined with a boolean in silico model. Mol. Oncol..

[27] Smyrek I., Mathew B., Fischer S.C., Lissek S.M., Becker S., Stelzer E.H.K. (2019). E-cadherin, actin, microtubules and FAK dominate different spheroid formation phases and important elements of tissue integrity. Biol. Open.

[28] Stadler M., Scherzer M., Walter S., Holzner S., Pudelko K., Riedl A., Unger C., Kramer N., Weil B., Neesen J. (2018). Exclusion from spheroid formation identifies loss of essential cell-cell adhesion molecules in colon cancer cells. Sci. Rep..

[29] Miranti C.K., Brugge J.S. (2002). Sensing the environment: A historical perspective on integrin signal transduction. Nat. Cell Biol..

[30] Saias L., Gomes A., Cazales M., Ducommun B., Lobjois V. (2015). Cell-cell adhesion and cytoskeleton tension oppose each other in regulating tumor cell aggregation. Cancer Res..

[31] Yoshii Y., Waki A., Yoshida K., Kakezuka A., Kobayashi M., Namiki H., Kuroda Y., Kiyono Y., Yoshii H., Furukawa T. (2011). The use of nanoimprinted scaffolds as 3D culture models to facilitate spontaneous tumor cell migration and well-regulated spheroid formation. Biomaterials.

[32] Meng W., Takeichi M. (2009). Adherens junction: Molecular architecture and regulation. Cold Spring Harb. Perspect. Biol..

[33] Stehbens S., Wittmann T. (2012). Targeting and transport: How microtubules control focal adhesion dynamics. J. Cell Biol..

[34] Mitra S.K., Hanson D.A., Schlaepfer D.D. (2005). Focal adhesion kinase: In command and control of cell motility. Nat. Rev. Mol. Cell Biol..

[35] Tancioni I., Miller N.L.G., Uryu S., Lawson C., Jean C., Chen X.L., Kleinschmidt E.G., Schlaepfer D.D. (2015). FAK Activity protects nucleostemin in facilitating breast cancer spheroid and tumor growth. Breast Cancer Res..

[36] Thakur R., Trivedi R., Rastogi N., Singh M., Mishra D.P. (2015). Inhibition of STAT3, FAK and Src mediated signaling reduces cancer stem cell load, tumorigenic potential and metastasis in breast cancer. Sci. Rep..

[37] Liu Q., Cheng C., Huang J., Yan W., Wen Y., Liu Z., Zhou B., Guo S., Fang W. (2024). MYH9: A Key Protein Involved in Tumor Progression and Virus-Related Diseases. Biomed. Pharmacother..

