# Supplementary material for: Proteomic Analysis Reveals Cadherin, Actin, and Focal Adhesion Molecule-Mediated Formation of Cervical Cancer Spheroids
Source: Cells. 2024 Dec 4;13(23):2004. doi: 10.3390/cells13232004 (PMC11640617; doi:10.3390/cells13232004)
Supplement: Supplementary file 1 [file cells-13-02004-s001.zip › cells-3283379-supplementary.pdf]

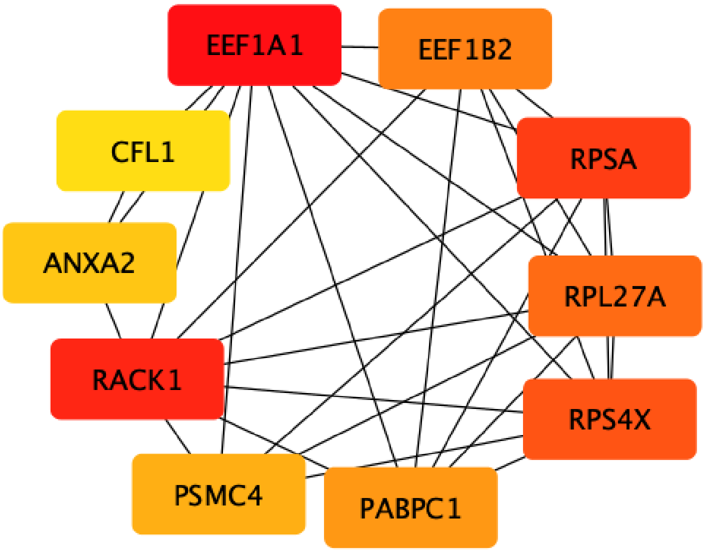

Figure S1. Subnetwork of the top 10 hub proteins in the protein–protein interaction network of upregulated proteins in 3D-HeLa spheroids. The color of node reflects the degree of connectivity. The pseudocolor scale from red to yellow represents the top nine hub protein ranks from 1 to 10. Red, orange, and yellow represent the highest, intermediate, and lowest degrees, respectively.

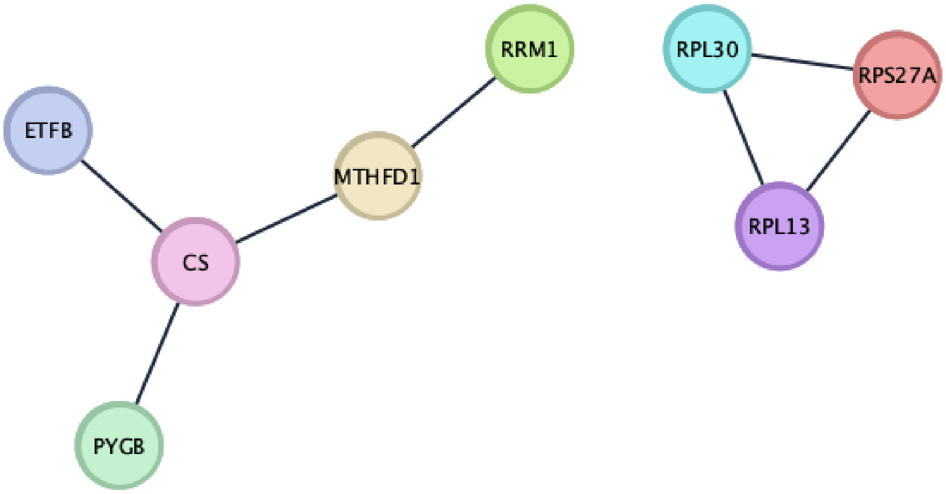

Figure S2. Protein–protein interaction networks of eight significantly upregulated proteins in the 3D-SiHa spheroid group.

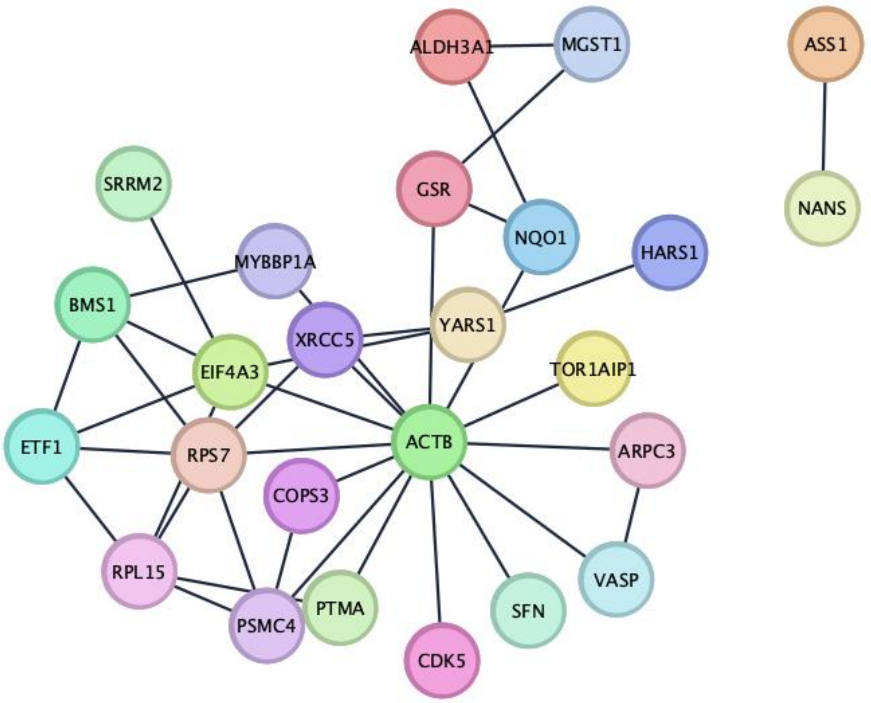

Figure S3. Protein–protein interaction networks of 29 significantly upregulated proteins in the 3D-C33A spheroid group.

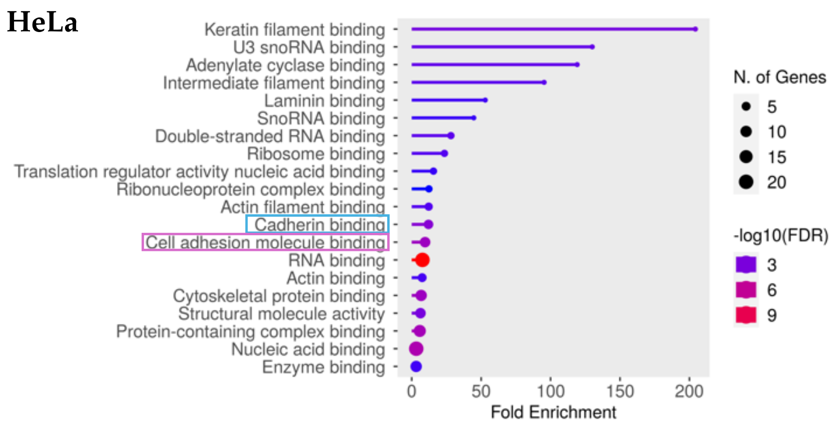

(a)

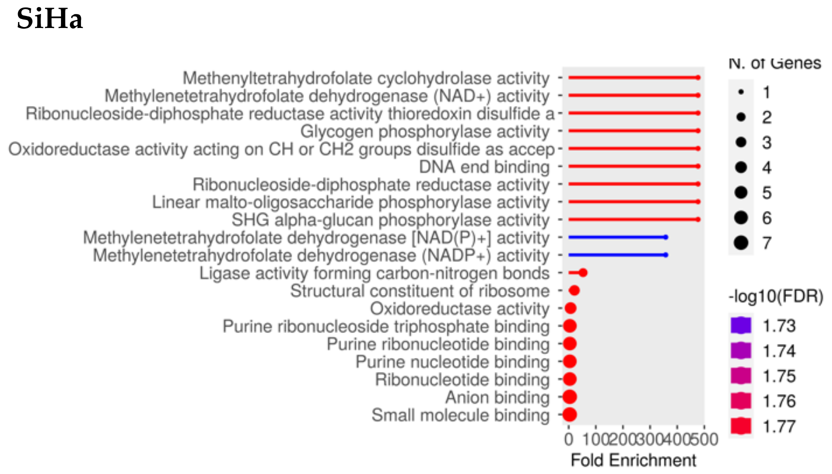

(b)

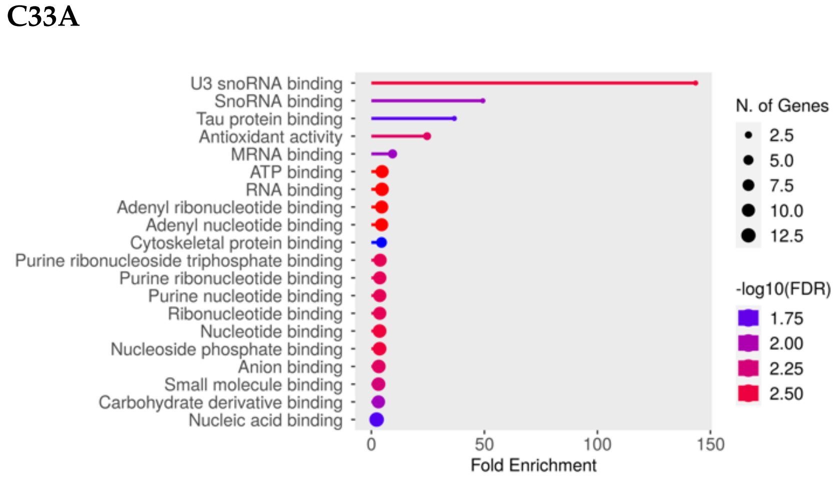

(c)

Figure S4. Gene ontology (GO) analysis of molecular function using the ShinyGO tool (v.0.77). (**a**), (**b**), and (**c**) represent the significant molecular functions for upregulated genes in 3D-HeLa, 3D-SiHa, and 3D-C33A spheroids, respectively. Adjusted *p*-value cut-off <0.05.

HeLa

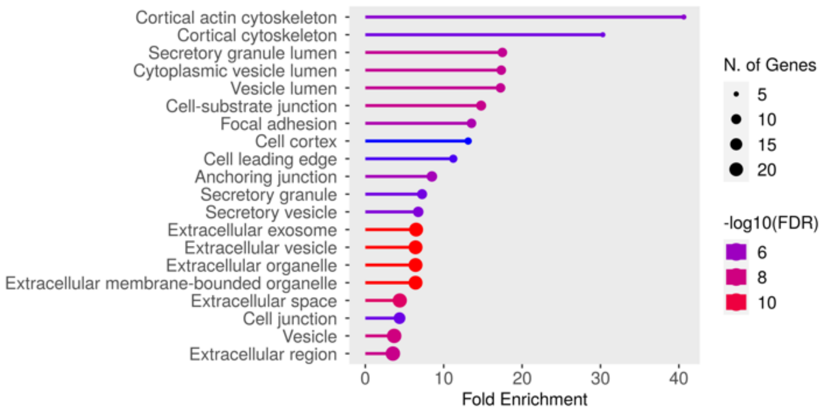

(a)

SiHa

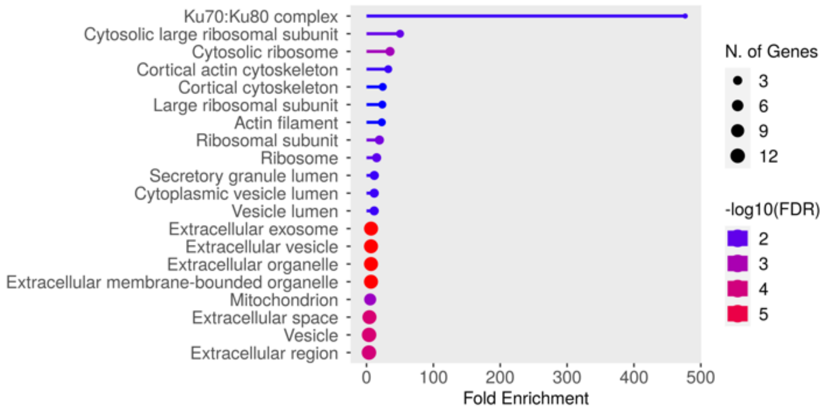

(b)

C33A

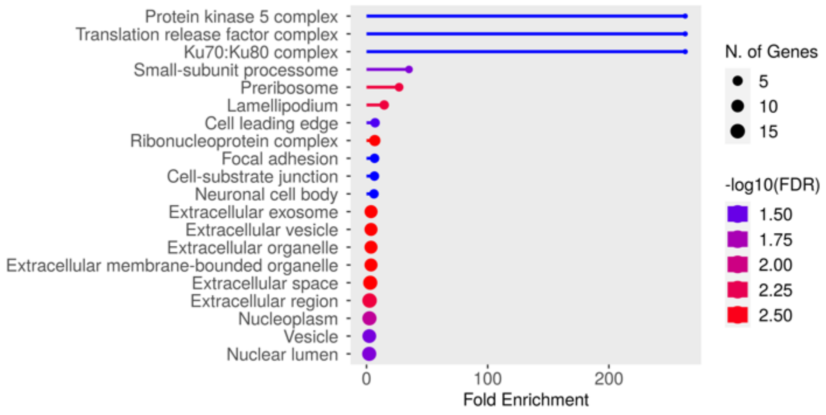

(c)

Figure S5. Gene ontology (GO) analysis of cellular component using the ShinyGO tool (v.0.77). (**a**), (**b**), and (**c**) represent the significant cellular component terms for upregulated genes in 3D-HeLa, 3D-SiHa, and 3D-C33A spheroids, respectively. Adjusted *p*-value cut-off <0.05.

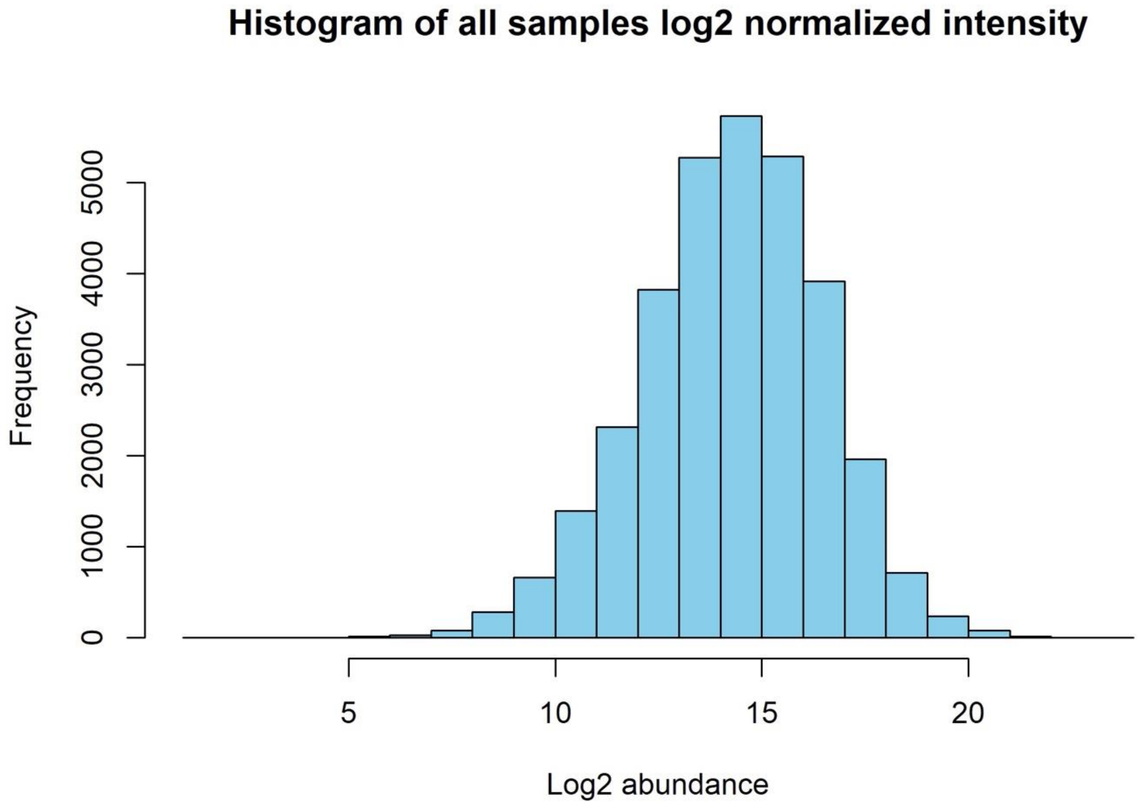

Figure S6. The histograms of normalized abundance values of all samples.

Table 1S. Total proteins identified from mass spectrometry.

number of identified proteins			
**Cell line**	**replicante (n)**	**2D**	**3D**
HeLa	1	1003	1004
	2	1003	600
	3	1004	1000
SiHa	1	1004	623
	2	1003	633
	3	635	1003
C33A	1	1004	1003
	2	998	1004
	3	1004	860

Table 2S. Downregulated proteins in 3D-HeLa spheroids with $log_2FC < -1$ and $p < 0.05$.

No.	protein_name	protein_id	logFC	P.Value
1	IF16	Q16666	-2.2885	0.0013
2	MACF1	Q9UPN3	-3.0069	0.0015
3	SYUA	P37840	-2.4707	0.0023
4	ERO1A	Q96HE7	-2.6231	0.0026
5	FETUA	P02765	-2.7441	0.0028
6	K22E	P35908	-2.9240	0.0040
7	ISG15	P05161	-2.7769	0.0040
8	DDX46	Q7L014	-2.4518	0.0045
9	SRSF2	Q01130	-1.9554	0.0072
10	IPYR	Q15181	-2.6311	0.0081
11	SP16H	Q9Y5B9	-2.7129	0.0085
12	HBA	P69905	-3.2933	0.0099
13	CHM1A	Q9HD42	-2.5820	0.0101
14	MIC19	Q9NX63	-3.6248	0.0103
15	EIF3J	O75822	-1.7786	0.0106
16	COPD	P48444	-1.5566	0.0112
17	INF2	Q27J81	-2.4778	0.0117
18	PEPL1	Q8NDH3	-2.0800	0.0124
19	GTF2I	P78347	-1.7913	0.0126
20	RLA2	P05387	-3.0467	0.0136
21	TRFL	P02788	-3.2714	0.0146
22	RAGP1	P46060	-1.7960	0.0168
23	ODPX	O00330	-2.0541	0.0169
24	PTGR1	Q14914	-2.3485	0.0176
25	PML	P29590	-1.5683	0.0184
26	K1C10	P13645	-1.8790	0.0188
27	SC31A	O94979	-2.3059	0.0192
28	TRI29	Q14134	-2.0397	0.0194
29	VASP	P50552	-1.7127	0.0207
30	SRSF6	Q13247	-2.3130	0.0223
31	VTDB	P02774	-2.1359	0.0226
32	K2C1	P04264	-2.3046	0.0228
33	GHITM	Q9H3K2	-1.8687	0.0235
34	SRSF9	Q13242	-1.5984	0.0247
35	TRA2B	P62995	-2.0683	0.0249
36	BCLF1	Q9NYF8	-2.5106	0.0250
37	NOTC3	Q9UM47	-2.5632	0.0274

38	SRP09	P49458	-1.4956	0.0299
39	LMNB2	Q03252	-2.0292	0.0305
40	PSME2	Q9UL46	-2.6154	0.0320
41	PRPF3	O43395	-1.8957	0.0331
42	H2A1J	Q99878	-2.4263	0.0331
43	THIL	P24752	-1.7274	0.0370
44	QOR	Q08257	-2.2127	0.0370
45	MAOM	P23368	-2.2217	0.0370
46	NAA50	Q9GZZ1	-1.8305	0.0376
47	FUBP3	Q96I24	-2.1372	0.0394
48	GBB2	P62879	-2.9501	0.0409
49	ETFA	P13804	-2.3892	0.0411
50	STAU1	O95793	-1.5410	0.0430
51	IF2B	P20042	-1.6699	0.0447
52	NAMPT	P43490	-1.7967	0.0456
53	COX5B	P10606	-1.7946	0.0474
54	CLCA	P09496	-1.8837	0.0495
55	RUVB2	Q9Y230	-1.3815	0.0497

Table 3S. Downregulated proteins in 3D-SiHa spheroids with log_2FC <-1 and $p < 0.05$.

No.	protein_name	protein_id	logFC	P.Value
1	IF4G2	P78344	-4.3898	0.0017
2	MCM2	P49736	-2.2121	0.0026
3	IPO5	O00410	-3.1835	0.0029
4	INF2	Q27J81	-2.9217	0.0039
5	SP16H	Q9Y5B9	-3.0373	0.0039
6	TRA2B	P62995	-2.7618	0.0041
7	SGTA	O43765	-3.1620	0.0042
8	DDAH2	O95865	-4.1164	0.0044
9	HPRT	P00492	-1.7080	0.0049
10	ERO1A	Q96HE7	-2.6736	0.0052
11	PSB7	Q99436	-2.6042	0.0078
12	GTF2I	P78347	-1.9247	0.0080
13	DPYL2	Q16555	-2.3142	0.0085
14	ERH	P84090	-2.2105	0.0146
15	EF1G	P26641	-2.4288	0.0149
16	SYFA	Q9Y285	-3.1116	0.0216
17	DDX17	Q92841	-2.2350	0.0234
18	AIFM1	O95831	-2.0404	0.0244
19	SODC	P00441	-2.5242	0.0265
20	RL35A	P18077	-1.7271	0.0279
21	ADRM1	Q16186	-4.4530	0.0294
22	NAMPT	P43490	-2.6585	0.0295
23	AL1B1	P30837	-1.5513	0.0310
24	SYMPK	Q92797	-3.1727	0.0321
25	A2MG	P01023	-2.3256	0.0332
26	EIF3E	P60228	-4.1623	0.0338
27	NUDC	Q9Y266	-2.7789	0.0341
28	STRAP	Q9Y3F4	-1.7966	0.0351
29	PSD13	Q9UNM6	-1.6125	0.0368
30	ODPX	O00330	-1.7281	0.0404
31	PSA2	P25787	-3.7701	0.0405
32	PDXK	O00764	-4.4560	0.0405
33	F10A1	P50502	-2.3416	0.0410
34	TMX1	Q9H3N1	-2.3694	0.0415
35	ECHA	P40939	-2.1884	0.0416
36	EDC4	Q6P2E9	-2.5827	0.0419
37	COF2	Q9Y281	-2.1290	0.0428

38	CLU	O75153	-1.7703	0.0481
39	RAB5C	P51148	-1.4447	0.0495

Table 4S. Downregulated proteins in 3D-SiHa spheroids with $log_2FC < -1$ and $p < 0.05$.

No.	protein_name	protein_id	logFC	P.Value
1	NUCKS	Q9H1E3	-2.4821	0.0106
2	RALY	Q9UKM9	-2.0985	0.0455
3	G3BP2	Q9UN86	-1.4290	0.0483
